# Support Provided by Caregivers for Community-Dwelling Diabetic Hispanic Adults with Intellectual Disabilities and Comorbid Conditions

**DOI:** 10.3390/ijms24043848

**Published:** 2023-02-14

**Authors:** Priyanka Rawat, Ujala Sehar, Jasbir Bisht, P. Hemachandra Reddy

**Affiliations:** 1 Department of Internal Medicine, Texas Tech University Health Sciences Center, Lubbock, TX 79430, USA; 2 Department of Pediatrics, Texas Tech University Health Sciences Center, Lubbock, TX 79430, USA; 3 Department of Speech, Language and Hearing Sciences, School Health Professions, Texas Tech University Health Sciences Center, Lubbock, TX 79430, USA; 4 Department of Public Health, School of Population and Public Health, Texas Tech University Health Sciences Center, Lubbock, TX 79430, USA; 5 Neurology Department, School of Medicine, Texas Tech University Health Sciences Center, Lubbock, TX 79430, USA; 6 Nutritional Sciences Department, College of Human Sciences, Texas Tech University, Lubbock, TX 79409, USA

**Keywords:** family caregivers, Hispanics, diabetes/obesity, prediabetes, lifestyle factors, impaired glucose tolerance

## Abstract

Diabetes is an age-related chronic health condition and a major public health concern. Diabetes is one of the significant causes of morbidity and mortality and a major contributing factor to dementia. Recent research reveals that Hispanic Americans are at an increased risk of chronic conditions such as diabetes, dementia, and obesity. Recent research also revealed that diabetes develops at least ten years earlier in Hispanics and Latinos than in neighboring non-Hispanic whites. Furthermore, the management of diabetes and providing necessary/timely support is a challenging task for healthcare professionals. Caregiver support is an emerging area of research for people with diabetes, mainly family caregiver support work for Hispanic and Native Americans. Our article discusses several aspects of diabetes, factors associated with diabetes among Hispanics, its management, and how caregivers can support individuals with diabetes.

## 1. Introduction

Diabetes is a chronic health condition affecting millions globally [1]. Diabetes is one of the significant reasons for mortality and morbidity [2,3]. Diabetes has worldwide societal implications, mostly in developing countries, where the beginning of diabetes at an early age can cause disability and socioeconomic costs [4]. Before developing type 2 diabetes, people usually have prediabetes which is also a serious health condition. When a person has elevated blood glucose levels but does not fulfil the diagnostic criteria for diabetes, it is known as prediabetes [5]. Diabetes is a prolonged health condition that affects blood glucose usage in the body. Insulin, a hypoglycemic hormone, is responsible for regulating blood glucose. Increased glucose concentration in the bloodstream is associated with increased appetite, thirst, and frequent urination [1]. Later that can lead to severe illnesses, including vision loss, kidney disease, heart disease, and lower limb amputation. The effective management of diabetes also depends on caregivers. Patients with diabetes must perform numerous everyday tasks to keep their condition under control. To complete each job, they frequently require the assistance of a committed caregiver. The daily tasks of a diabetic family member are shared by the caregiver, who also offers emotional support.

Lifestyle factors, including unhealthy dietary patterns, lack of physical exercise, stress, and obesity, are responsible for a diabetic condition. However, the primary cause of diabetes varies depending on the type. A diabetic condition can be delayed or prevented with healthy lifestyles such as physical activity, healthy diet, yoga, and meditation. Type 1 diabetes mellitus (T1DM) requires medication, while type 2 diabetes mellitus (T2DM) can be controlled by lifestyle factors; however, if lifestyle factors are ineffective, patients may require medication. Still, diabetic patients are troubled by several potentially fatal complications [6]. The psychosocial influence of diabetes in the early years is global and involves the entire family, schools, and society [7]. Individuals of a specific racial and ethnic group are more prone to developing prediabetes and T2DM.

According to the Centers for Disease Control and Prevention (CDC), Hispanic/Latino adults are 50% more likely to develop T2DM at a younger age. The possibility of having diabetes is closely related to their background. In the Hispanic community, socioeconomic factors, cultural and multiple medical factors impact the progress of diabetes, its course, and outcomes [8].

According to the CDC National Diabetes Statistics Report 2022, more than 130 million individuals are surviving with diabetes or prediabetes in the United States. An estimated 1.4 million new cases of diabetes among individuals 18 and older were found in 2019. Diabetes diagnosis rates among persons by race/ethnic background are as follows: American Indians/Alaskan Natives, 14.5%; non-Hispanic blacks, 12.1%; Hispanics, 11.8%; Asian Americans, 9.5%; and non-Hispanic whites (NHWs), 7.4%. Individuals with a household income that is less than the federal poverty line have an increased prevalence of diabetes, for men (13.7%) and women (14.4%). Considering the high prevalence of diabetes in the USA, this article talks about various aspects of diabetes, factors associated with diabetes among Hispanics, diabetes management, and the support caregivers provide for the community.

## 2. Difference between Hispanics and Non-Hispanics

According to the United States Census Bureau, the Hispanic ethnic group includes any individuals of Puerto Rican, Cuban, South/Central American, Mexican, and Spanish-speaking people, irrespective of race. Hispanic/Latino are pan-ethnic words aiming to explain and summarize the population of individuals of that ethnic background residing in the United States. According to the United States Census 2020 data, 62.1 million Hispanics reside in the United States, representing 18.9% of the total U.S. population. After non-Hispanic whites, Hispanics are the second largest and fastest-growing racial/ethnic group.

In 2022, Hispanic or Latino minority residents were projected to exceed non-Hispanic white (NHWs) as the majority population in Texas [9]. According to the United States Census Bureau, NHWs are European Americans, North African Americans, and Middle Eastern Americans. Non-Hispanic Latino is another term for NHWs. Americans of European origin represent multiple ethnic groups, and more than 50% of the white population are English, Scottish, Irish, German, French, Italian, and Polish Americans. Non-Hispanic whites have created their own music, fashion, art, cuisine, and political economy, mostly centered on an amalgamation of traditional European ones.

## 3. Type of Diabetes

**Figure 1:**
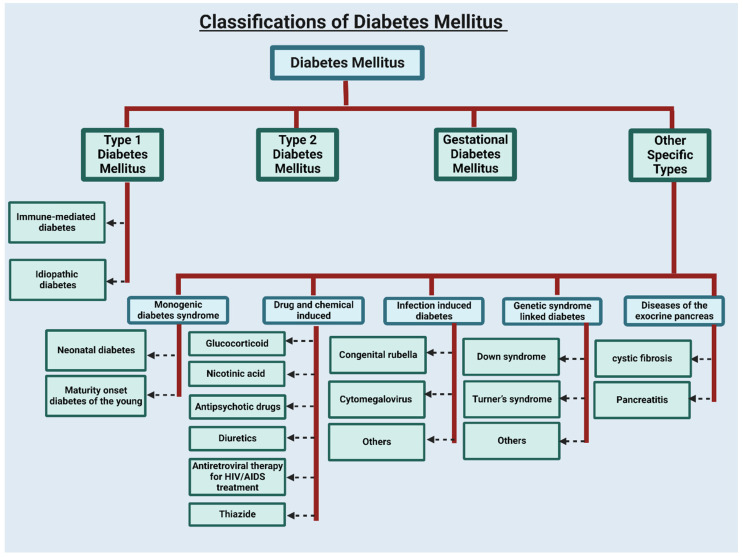
Classification of diabetes mellitus. Generally, diabetes can be categorized into four categories. Created with BioRender.com.

Diabetes is a diverse metabolic disorder and is challenging to classify [10]. Diabetes can be characterized into the following four categories: (a) type 1 diabetes, (b) type 2 diabetes, (c) specific types of diabetes, and (d) gestational diabetes [11] (refer to Figure 1).

Type 1 diabetes mellitus (T1DM): T1DM only represents around 10% of the cases globally [12] and generally occurs early in life, at approximately 30 years old or younger [1,10]. T1DM is an autoimmune condition that destroys insulin-producing beta cells, leading to hyperglycemia. [13]. T1DM can be detected at any age, but it is one of childhood’s most common chronic diseases [14]. The traditional definition of T1DM is “juvenile” diabetes; it can manifest at any age, with adulthood accounting for up to 50% of cases [15]. T1DM is also known as “insulin-dependent diabetes” due to the dependency of T1DM individuals on insulin shots to maintain blood glucose levels [1]. Children with T1DM generally showed symptoms of polydipsia, weight loss, polyuria, and diabetic ketoacidosis [16]. Men and boys are slightly more likely than women and girls to experience this [17]. T1DM is subdivided into idiopathic and immune-mediated DM [10].

Type 2 diabetes mellitus (T2DM): T2DM typically occurs late in life, appears in individuals aged 30 or older, and is known as late-onset DM [1,10]. T2DM is the most general form of diabetes. Because of pancreatic-cell dysfunction and insulin resistance in the target organs, T2DM is characterized by a relative insulin deficiency [18]. This reduces glucose transport into the liver, muscle, and fat cells [19]. T2DM is also known as adult-onset and insulin-resistant diabetes. Over 90% of the diabetic population is T2DM [1]. T2DM is primarily caused by lifestyle factors and genetics [20]. Most individuals with T2DM are obese, with abdominal obesity; consequently, the adipose tissue plays a central role in the pathogenesis of T2DM [19]. T2DM is characterized by increased hyperinsulinemia, impaired insulin sensitivity, and pancreatic beta-cell dysfunction, with up to 50% cell loss at diagnosis [21]. According to data published by WHO (Geneva, Switzerland), the occurrence of T2DM has significantly increased in countries of all income levels in the past three decades.

Specific type of diabetes: This category of diabetes includes a wide variety of uncommon conditions. This type of diabetes has monogenic diabetes syndrome (maturity-onset diabetes of the young and neonatal diabetes), drug and chemical-induced diabetes (use of a glucocorticoid, diuretics, a few HIV medications, thiazide, nicotinic acid, atypical antipsychotics drug and others), infection-induced diabetes (congenital rubella, Cytomegalo virus, and others), a genetic syndrome linked with diabetes (Down syndrome, Turner’s syndrome, and others), and diseases of the exocrine pancreas (cystic fibrosis and pancreatitis), etc. [11,22,23,24].

Gestational diabetes mellitus (GDM): Gestational diabetes develops in some females during pregnancy and is called glucose intolerance [25]. An estimated 5–7% of all pregnancies experience problems related to GDM, causing more than 200,000 cases yearly, but this rate can be increased due to obesity [12,26]. Evidence suggests that mild maternal hyperglycemia is a risk factor for fetus morbidity [27], but that morbidity occurs only in a few cases. It generally disappears after delivery but increases the risk of T2DM. GDM is linked with high blood pressure during pregnancy, known as preeclampsia, and premature birth. GDM increases the risk of having T2DM later on in life.

## 4. Development of Diabetes

Although the precise etiology of diabetes is unknown, environmental and genetic factors work together to promote the disease [6]. Researchers suggest that T1DM develops due to genes and environmental factors, including viruses, that might provoke the condition. Conversely, T2DM is caused by genes, lifestyle factors, diet, and physical inactivity [1]. Lifestyle risk factors or modifiable factors such as increased body mass index (BMI), poor nutrition, hypertension, physical inactivity, alcohol use, and smoking cause diabetes [28,29]. Psychosocial factors, including increased stress, depression, poor mental health status, and lower social support, also are linked with an elevated risk of developing diabetes [30,31,32,33].

## 5. Diabetes in Hispanic and Latino Communities

**Figure 2:**
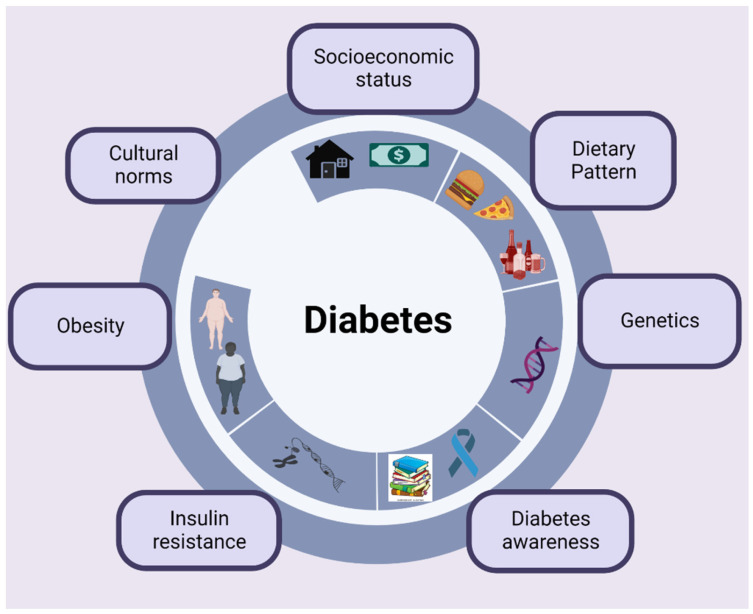
Factors associated with the occurrence of diabetes mellitus in Hispanics. Created with BioRender.com.

Hispanics share the same risk factor for T2DM as other ethnic groups. According to the report published by the CDC in 2022, an average of 17% of Hispanic or Latino individuals are expected to have T2DM. In the United States, adults have a 40% chance of developing T2DM, but Hispanic or Latino individuals have a more than 50% chance of developing T2DM. Several elements could contribute to the emergence of T2DM in the Hispanic or Latino community (refer to Figure 2). According to the CDC, Hispanics are at high risk of having diabetes due to obesity, family history with T2DM, and physical inactivity (exercising less than 3 times a week). Hispanics demonstrate worse self-management of T1DM compared to NHWs [34], and blood glucose self-monitoring is observed to be less common in Hispanics than NHWs [35]. As discussed above, the rates of diagnosed diabetes are higher in African Americans and Hispanics compared to Asian Americans and NHWs. The possible factors for higher diabetic rates in Hispanics are as follows:

Dietary pattern: Within the Hispanic community in the US, dietary choice, such as a diet that is rich in simple carbohydrates, is one of the significant risk factors for diabetes. Many factors including low socioeconomic status and acculturation are hurdles to healthy food preferences [36]. Hispanics generally have saturated-fat- and carbohydrate-rich food [37]. Studies have shown that acculturation in Hispanic American immigrants enhances dietary habits that encourage obesity, such as sugary beverages, higher sugar consumption, higher solid fat consumption, more fast food intake, and more eating out [38]. Cultural norms around the diet and less access to a nutritious diet are other primary reasons for diabetes among Hispanic people.

Insulin resistance: Hispanic individuals are more insulin resistant than non-Hispanic whites (NHWs), putting them at a higher risk of developing T2DM [39]. Regarding insulin resistance, Hispanic individuals are more likely to have a genetic predisposition for β cell impairment that fully manifests in the manifestation of insulin resistance, environmental factors, and other metabolic risk factors [8]. K.C. Chiu and colleagues’ study found that the prevalence of T2DM is higher in Hispanics than in the other three ethnic groups [40]. They also found that Hispanics were more insulin resistant than non-Hispanic whites [40].

Obesity: Many studies found a high rate of obesity is observed in Hispanics [41,42]. A surprising increase in the predominance of obesity in early life was observed in individuals of Hispanic origin [43]. According to the United States Department of Health and Human Services Office of Minority Health, 78.8% of Hispanic American women are obese or overweight. Cultural factors and socioeconomic factors are associated with obesity [44,45]. Lower socioeconomic status has been linked with poor health conditions and leads to higher rates of chronic disease, including diabetes, obesity, and cardiovascular disease among Hispanics [46]. Iris Shai and colleagues’ study found obesity as a fundamental risk factor for the development of diabetes among Hispanic women as compared to whites [47]. Matthew and colleagues discovered polymorphisms close to the SOCS3 gene associated with features related to obesity and glucose homeostasis in Hispanic Americans. Leptin and insulin signaling is inhibited by the SOCS3 gene product, which also participates in the response inhibition of various cytokine signals that result in insulin resistance [48].

Genetic susceptibility: Many studies showed the Hispanic population’s genetic propensity to obesity; various candidate loci, such as INSIG2, NGEF, and RGS6, have been connected with increased adipose tissue [49,50]. A.L. Williams and coworkers discovered sequencing variations in SLC16A11 and HNF1A genes. They found that the SLC16A11 gene mutation enhanced the chance of getting diabetes and accounts for around 20% of the rise in T2DM in Mexico. SLC16A11 was discovered through research in Latin Americans and Mexicans to be a novel candidate gene for T2DM with a putative function in triacylglycerol metabolism [51].

Lack of awareness: Awareness about diabetes is essential to changing behavior and lifestyle and may reduce the risk of developing diabetes. According to a study by Neil Schneiderman and colleagues, the Hispanic community has a high prevalence of diabetes and low rates of awareness of the disease. Additionally, they discovered a low level of health insurance, diabetes control, and awareness, along with a negative correlation between diabetes and household income and education. This relation remained noteworthy even after considering factors such as age, BMI, sex, field center, Hispanic heritage, and length of time spent in the United States [52].

Genetic mutations: Genetic mutations and/or single nucleotide polymorphisms (SNPs) are higher in American Indians/Alaskan Natives, followed by African Americans and Hispanics. Single genes cause diabetes resulting in a decreased production of insulin hormone in the beta cells [1]. Global research on the genetics of diabetes has focused on SNPs and gene mutations in nuclear and mitochondrial DNA as well as in different ethnic groups [53]. Amanda and colleagues’ pilot study on SNPs found that 26 SNPs are linked to T2DM in Hispanic adults [54]. In addition, they found these 26 SNPs had not been reported to be connected with T2DM earlier. Additionally, among the 26 SNPs, many of them were sited close to those genes that had been earlier linked with diabetes [54].

## 6. Complications Due to Diabetes

Diabetes is particularly concerning because it gradually increases the prevalence of other chronic and acute diseases in the population, having a significant impact on quality of life, demand for public health services, and monetary costs [55]. Uncontrolled diabetes can lead to a metabolic imbalance that brings serious problems that need to be treated immediately. Increased intracellular glucose levels are believed to enhance reactive oxygen species (ROS) production and alter a sequence of critical downstream pathways, including activating protein kinase c, polyol pathway flux, hexosamine pathway flux, and the formation and activation of advanced glycation end products [56].

**Figure 3:**
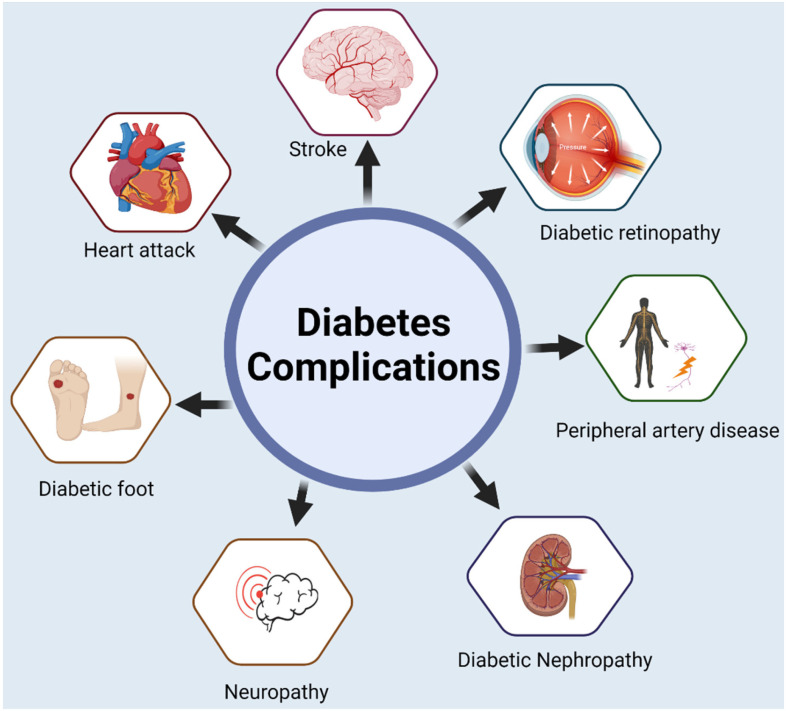
Diabetes affects multiple body functions and leads to complications such as stroke, diabetic retinopathy, heart disease, foot ulcer, and kidney disease. Created with BioRender.com.

The burden associated with diabetes is caused by both macrovascular complications, such as stroke and peripheral vascular disease, coronary heart disease, and microvascular complications, such as end-stage renal disease (ESRD), neuropathy, and diabetic retinopathy along with lower-extremity amputations (LEA) [55] (refer to Figure 3). Amputation may be necessary due to gangrene, bruising, or wounds that do not heal due to peripheral vascular disease [5]. Due to these complications, there is an increased risk of hospitalization and caregiver burden. Emerging evidence suggests that diabetic patients are at higher risk of developing anxiety [57], eating disorders (especially in younger females with T1DM) [58], depression [59,60], and severe mental illness [61]. Diabetes-related lower extremity amputations have been observed to occur at an abnormally higher rate in Hispanics than in Whites [62].

In their study, Andrea Cherrington and colleagues discovered that the difficulties in managing diabetes in Hispanic people led them to feel anxious, hopeless, and upset. Additionally, they explained why people lack the will to collaborate with family members, get out of bed, work, and practice self-care behaviors [63].

## 7. Acute Complications

Acute complications result from uncontrolled blood glucose levels that are too high (hyperglycemia) or too low (hypoglycemia), which might be due to mismatching the required need for insulin. Diabetic ketoacidosis, hyperosmolar hyperglycemic nonketotic coma, and hypoglycemia come under acute complications [64]. Uncontrolled diabetes can lead to a metabolic imbalance that results in acute complications and needs immediate attention.

Hyperosmolar hyperglycemic nonketotic syndrome: High blood glucose levels provoke the frequency of urination and lead to severe dehydration in individuals with diabetes [65]. This syndrome is described by severe hyperglycemia, a significant surge in serum osmolality, and clinical indications of dehydration lacking the significant deposition of ketoacids [66]

Diabetic ketoacidosis: According to the CDC, due to the improper function of insulin, the body cell gets starved of glucose even with high blood glucose levels. Under this condition, cells start using body fat as an energy source; as a result, liver cells generate ketone bodies from fatty acids. Brain cells can use ketone bodies in place of free fatty acids when blood glucose levels are low. However, ketone concentrations that are too high can cause urine to become acidic, which can cause prolonged unconsciousness or even death [65].

## 8. Chronic Diabetic Conditions

**Figure 4:**
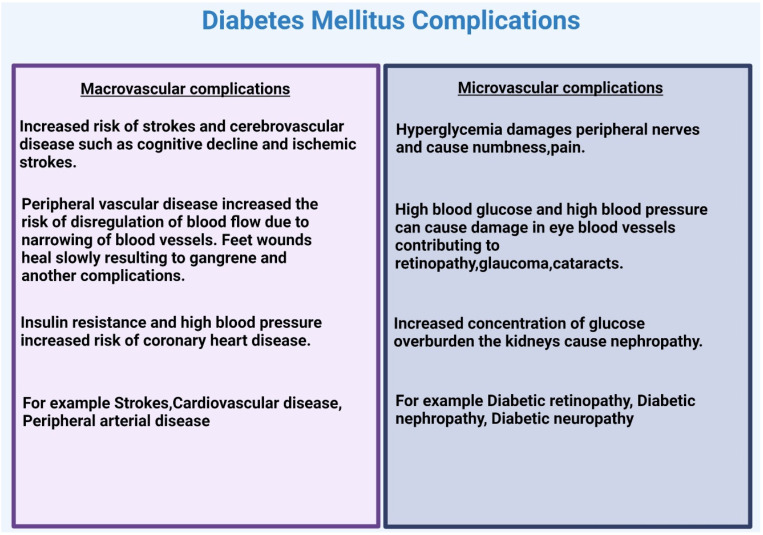
Long-term diabetic complications can result in microvascular and microvascular problems. Diabetic patients and their immediate care. Created with BioRender.com.

Prolonged diabetes can cause microvascular and microvascular complications (refer to Figure 4). These chronic conditions need constant monitoring and treatment.

Macrovascular complications: Various suggestions have been proposed to describe the process of developing microvascular complications, comprising the formation of reactive oxygen species and oxidative stress, the production of end products from advanced glycation, the stimulation of the polyol pathway induction of flux through the hexosamine pathway, the triggering of protein kinase C, and altered expression and activity of growth factors [67]. Macrovascular complications affect large blood vessels [68]; these vessels transport blood to the brain, heart, and extremities. High concentrations of glucose in the blood (hyperglycemia) cause protein glycation. Protein glycation is when protein is exposed to a high sugar level for a long time, and by the nonenzymatic process, it may attach to proteins [69]. The process of glycation, lipid deposition, inflammation, and other factors cause the narrowing of blood vessels, which raises the risk of cardiovascular disease, stroke, and the need for amputation of the extremities. Peripheral vascular disease due to diabetes is caused by narrowing blood vessels and an increased risk of dysregulation of blood transport in the legs [68]. Feet wounds heal slowly and cause gangrene and other complications. People with diabetes are unable to feel pain due to nerve loss and numbness; therefore, foot ulcers may lie unnoticed for a long time. In severe situations, the infection and ulcer may cause gangrene and necessitate amputations. Larger brain blood arteries are also impacted by diabetes, which raises the risk of stroke and other cerebrovascular illnesses such as cognitive impairment and transient ischemic attack. Several cardiac disorders are made more likely by diabetes. The risk of coronary heart disease is increased by high blood pressure and insulin resistance. Major vascular harm may prompt myocardial dead tissue, stroke, rheumatoid joint pain, osteoporosis, and degenerative maturing [69].

Microvascular complications: The smaller blood vessels are affected by microvascular problems [68], which in some cause retinopathy, nephropathy, and neuropathy; individuals with impaired blood glucose levels have at least one of these complications in the phases of the disease [65,68].

Due to the demands of sticking to the patient’s healthcare regime, diabetes mellitus is a chronic ailment, and the family needs to make considerable behavioral adjustments. These expectations are linked to psychological conflicts between the patient and the family environment [70]. Beyond only maintaining adequate glucose control, patients with diabetes must also avoid complications, minimize their damage, and receive rehabilitation. The basic self-care practices for diabetic individuals who want positive outcomes include a nutritious diet, exercise, medication adherence, blood sugar monitoring, strong problem-solving skills, healthy coping strategies, and risk-reduction practices [71].

The primary source of care for elderly persons in need is their spouses [72]. They are usually in charge of most elderly persons with disabilities and offer the most thorough and intensive care. Additionally, spouses take on the caregiver position for longer than other caregivers, are more likely to be care managers than providers, and can tolerate higher levels of handicap. However, their older age and associated chronic health issues put their caregiving spouses at risk of severe physical and mental health issues [73].

To investigate the support provided by professional caregivers in community living situations to diabetic adults with mild to severe intellectual disabilities, thirteen caregivers took part in semi-structured interviews for a qualitative study. Most professional caregiver assistance in diabetes care is focused on managing medication administration and dietary restrictions. To offer person-centered care, caregivers must balance preserving a client’s health with their need to uphold autonomy. The study’s results revealed that none of the caregivers had training in assisting self-management and had just a basic understanding of diabetes. The few practitioners that encouraged client self-management emphasized the significance of a supportive and cooperative approach [74]. The effective management of diabetes depends heavily on caregivers. Patients with diabetes must perform numerous everyday tasks to keep their condition under control. Support from a dedicated caregiver to perform routine tasks and diabetes management can help the quality of patients’ life significantly.

## 9. Diabetes Management

Diabetes cannot be life-threatening if it is managed properly. One can manage diabetes by adopting a healthy lifestyle, monitoring blood glucose levels, and adhering to medication. Since diabetes can affect almost every organ of the body, managing diabetes is crucial. Diabetes management can be accomplished in different ways. Self-care management is one way, and the other is usually in the case of older and young diabetic patients, where caregiving is required to support diabetics.

## 10. Self-Care Management

Approximately 90% of patients have T2DM among all diabetes mellitus patients [75]. T2DM patients are at significant risk of acquiring diabetes-related complications, including retinopathy, renal disease, and cardiovascular disorders. Global spending on diabetes mellitus (T2DM) and its consequences’ direct health care costs are anticipated to be between 997 and 1788 billion international dollars in 2040 [76]. The roots of diabetic treatment and the most difficult facets of self-management, food, and exercise are expected to be promptly integrated by T2DM patients to avoid diabetic complications [77].

Self-management entails different sets of activities that include medical management, such as taking medication and following dietary recommendations; behavioral management, such as adopting new behaviors in the context of chronic disease; and managing emotional stress, such as handling the frustration, fright, and despair associated with chronic disease [78]. Training and education on diabetes self-management have been found to enhance glucose control, potentially lowering the risk of long-term problems. In this age of technology, there are endless possibilities for using digital tools to teach diabetic individuals how to self-manage their disease with the growing popularity of cell phones and the Internet. Patients can use numerous websites, internet portals, and mobile applications to enhance their diabetes care. Studies on its efficiency and cost–benefits in managing diabetes, however, are scarce [79].

In recent years, there has been a substantial shift in diabetes education. Programs for diabetes self-management education now have a more considerable emphasis on providing continuing assistance to help patients maintain the improvements in self-management they have made because of education. These programs are now more patient-centered and theoretically oriented [80]. A study examined whether demographic or disease-specific factors influenced patients’ preferences by asking diabetes patients receiving care in public hospitals about their interest in various forms of self-management support. It has been discovered that race/ethnicity, linguistic proficiency, and self-reported health literacy all affect choices for self-management support delivery options. In safety-net situations, many diabetic patients indicate interest in receiving self-management support [81].

## 11. Caregiving in Diabetes

The two most common types of diabetic patients have different needs; T1DM in younger people requires assistance with glucose monitoring, insulin shots, and maintaining healthy lifestyles. In addition, T2DM patients, especially older people with diabetic complications, might need help monitoring glucose, treatments, mobility, and rehabilitation. Professional caregivers should meet such needs of people with diabetes, but in Hispanic families, the primary caregivers are family members with less or no knowledge of diabetes management.

Caregiving to young diabetic patients (YDP): One of the common chronic conditions in children, T1DM, has more than doubled in prevalence over the previous ten years. The occurrence varies across races, nations, and areas [82]. Parents and their children, who are at a crucial physical and psychological stage of development, find it incredibly difficult to manage their children’s T1DM since it is a time-consuming and demanding care task [83]. Caretakers frequently experience post-traumatic and parental stress, which impacts the child’s state and leads to increased degrees of psychopathology and poor treatment compliance [84]. Pediatric patients’ parents are bothered by several issues concerning treating their child’s illness. Throughout the challenging daily management process, this feeling continues. Parents are constantly concerned about their children’s hypoglycemia and other T1DM emergency circumstances [83,85,86,87].

A growing amount of qualitative research has observed children’s engagement in treating their diabetes alongside parents and an awareness that children are socially competent agents in their own right [83]. Young people have been the focus of many technological advancements, and in the past ten years, diabetes management has started to move toward technology-based care [88]. Early disease management requires care coordination across multiple disciplines between the diabetic child, parents, family, medical staff, and other caregivers such as teachers [89].

The parental response and the coping mechanisms to handle the difficulties of caring for a sick child depend on the type and time of the condition. Previous studies have demonstrated that parents are highly susceptible to health inequalities [90]. Stallwood and colleagues investigated the effects of caregiver burden, perceived stress, and coping strategies by managing and controlling blood glucose of young children with T1DM in the comfort of their homes. According to the study’s findings, lower Hgb A1c levels were linked to higher caregiver stress, and lower Hgb A1c was also linked to higher levels of home management. There was no discernible connection between caregiver coping and house management. Parents dealing with their child’s T1DM reported feeling stressed, and those who could keep their child’s glycemic management under control reported feeling more stressed. Despite the child’s apparent glycemic control, the study suggests that healthcare professionals should assess caregiver stress [91].

Children who live in poverty face risks from the environment, inadequate health insurance, and a decline in their physical, socioemotional, and cognitive well-being [92]. The diabetes epidemic disproportionately burdens Hispanics in the United States. According to the CDC, Hispanics have a 130% greater prevalence of diabetes than non-Hispanic whites [93]. Understanding these discrepancies is crucial, given the expansion of the Hispanic population and the rise in the frequency and incidence of T1DM among Hispanic children [94,95,96]. Although there is more material on the distinct healthcare experiences of the Hispanic community, few qualitative studies have reported on Hispanic kids with T1DM [96].

A recent study reported the perspectives of Hispanic T1DM patients and their Hispanic caregivers [97]. The study highlighted some significant issues Hispanic caregivers faced while taking care of T1DM patients. Hispanic caregivers acknowledged a range of nutrition-related worries, particularly with the foods they prepare and their cultural eating habits. Most participants agreed on the need to adhere to the patient’s therapy. Another critical issue among caregivers was a feeling of social isolation; they reported receiving little assistance from other family or community members due to cultural beliefs about the disease. Parents also acknowledged a dearth of knowledge and trust in their relatives’ capacity to manage their diabetes. Lastly, concerns about diabetes technology were the subject of worry derived from the survey participants’ experiences. Participants also said they did not think their children were ready for the technology. Many parents described their children’s fear of any overt physical manifestations of diabetes. The use of the pump and constant blood glucose monitoring has been associated with income, race/ethnicity, insurance status, language, and parent education in prior studies, which have further demonstrated disparities in the use of diabetes technology. [98,99,100].

Parents’ anxiety about their child’s diabetes was higher among low-income families of TIDM patients. There were notable racial disparities among caregivers, with Hispanic families frequently citing “recognizing the importance of family” as a component influencing their caregiving experience [101]. A study by Mello and colleagues looked into ethnic disparities in stress and coping in T1DM teenagers and their mothers [102]. The study discussed the intricate mechanisms that may be behind racial disparities in stress and coping related to diabetes in young adolescents and their mothers. The study’s findings expanded earlier research into Latino communities, where family engagement is an essential cultural value. They demonstrated that dyadic and individual stress and coping mechanisms are involved in managing diabetes-related stress during adolescence. It has been suggested that clinicians dealing with ethnically varied populations may find it beneficial to be aware that mothers may not completely comprehend the stressful aspects of diabetes for their adolescents and that this may be especially true in Latino homes. Adolescents and mothers may be able to establish a shared awareness of the numerous issues that an adolescent encounters when treating T1DM, by participating in a talk or requesting that families fill out a stress-related diabetes questionnaire.

Caregiving to adult diabetic patients (ADP): Because diabetes mellitus is a chronic condition that needs constant care, family caregivers are crucial in giving long-term care to those with it. The increased strain on family caregivers impacts the health of those with this disease. People with diabetes mellitus have a better health status when family caregivers are not under as much stress. Therefore, there is a need for interventions to improve diabetic patient health and reduce caregiver stress [103].

Although family caregivers play a significant role in managing medication, there are a few facts concerning the factors contributing to this concern and how this knowledge may be used to incorporate caregivers into the healthcare system. A study by Noureldin and colleagues showed that the participation of caregivers in various drug management activities was linked to a diversity of demographic and caregiving attributes. There were several recurring characteristics, including race and ethnicity, the particular clinical conditions of the patients receiving care, and caregiver involvement in assisting daily and medically relevant activities. These results can aid practitioners in educating and assisting caregivers more successfully. Healthcare providers can take the initiative to engage caregivers in discussions regarding medication management [104].

It has been established that caregiver stress harms their health, particularly for those who already have cardiovascular disease. With diabetes, a condition requiring a high level of self-care, little is known regarding the relationship between caregiver stress and health behaviors among caregivers. Diabetes self-care was evaluated using a composite self-care score that assessed performance across the four areas of Mediterranean diet adherence, smoking status, physical activity, and medication observance to investigate the relationship between diabetic self-care and caregiver stress among those with the disease. The findings of the study on adult diabetes caregivers in the US showed that low medication adherence was linked to high levels of caregiver stress. There appears to be a need for healthcare providers and caregivers to become more aware of the incidence of caregiver burden and its possible effects on caregivers’ self-care [105].

## 12. Trends of Diabetes Caregiving in Hispanics

America’s appearance is changing dramatically in terms of population. The ethnic group with the quickest rate of growth in the USA is Latinos/Hispanics. According to the 2020 Census, 62.1 million Hispanics were living in the United States. This demographic accounts for 18.9% of the U.S. population, making it the second-largest racial/ethnic group in the nation after non-Hispanic whites. It is typical in Hispanic culture to prioritize the family’s needs over those of the individual. This cultural practice, known as familismo or familism, affects how family caregivers care for their loved ones who are older adults. Systematic research in the Hispanic population focused on how family and familism beliefs affect self-care behaviors in treating diabetes [106,107]. In the US, the risk of diabetes is a significant worry, and it is on the verge of a pandemic in areas like the Southwest and Texas, where Mexican Americans make up most of the minority population [108]. Reviews looked at T2DM prevalence in Hispanic populations and found dangerous rates that are high compared to other racial and ethnic groups [109,110].

Compared to white caregivers (35%), 49% of Hispanic/Latino caregivers report heavier caregiving burdens. They devote more time to caregiving than their white counterparts (26 h for Hispanics/Latinos against 21 h for whites) and are more likely to assist their loved ones with nursing or medical activities (67% for Hispanics/Latinos vs. 52% for whites) [111].

Mexican culture’s central familism customs had positive and negative effects on managing diabetes. Setting up distinctive educational programs that involve family and community members and personalizing clinical care is necessary to improve the health of this population. Enhancing communication between clinicians and patients and fostering a better understanding can be accomplished by acknowledging and accepting the social stigma associated with diabetes in this population [112]. T2DM and its consequences affect people from ethnic minority communities more frequently than non-Hispanic persons. Although the precise causes of the discrepancies in diabetes prevalence between races and ethnic groups are unknown, it is likely due to a blend of inherited and acquired variables [35]. The sociocultural elements that affect diabetes management techniques must be considered in clinical practice (e.g., involving the family, particularly the principal female caregiver, and establishing home-based as well as community-based education sessions). Future research should concentrate on population-defined health and disease self-management, innovative educational interventions, and family and community treatments centered on the concept of the social stigma of illness to further minimize health inequalities in this population [112].

A study was performed in an urban emergency department with mostly diabetic Latino patients to describe the baseline level of disease-specific knowledge among these patients and the family members of these patients and to clarify the traits that are linked to greater diabetes knowledge. In the predominantly Latino urban emergency department patient population, diabetes-specific information was lacking in both patients and primary family caregivers, underscoring the need for more education in unconventional settings. Considering the findings, it was suggested that this education should concentrate on topics such as food, hyper- and hypoglycemia symptoms, and wound care. This study is the basis and reason for developing efficient and targeted instructional materials for emergency departments, enhancing patient knowledge and well-being [113].

## 13. Conclusions and Future Directions

The Hispanic community is the most significant and growing population in the United States, and Hispanic individuals are at high risk for diabetes. Many factors, such as educational, socioeconomic, cultural, and linguistic barriers, lifestyle, access to health care, higher insulin resistance, and genetic susceptibility to obesity, lead to the development of diabetes in the Hispanic community. Prolonged diabetes causes many complications, and patients have demands beyond just maintaining reasonable glucose control, including limiting their impairment, preventing complications, and getting rehabilitation. Since diabetes mellitus is a chronic condition that needs constant care, family caregivers in Hispanic families are crucial in offering long-term care for those with it. The health system of the United States is not sufficiently equipped to manage diabetes, especially in ethnic groups such as Hispanics. Specific interventions can help ethnic minorities such as Hispanics to improve their lifestyle by managing diabetes effectively, such as education, outreach, research and development, technological interventions, and culturally acceptable clinical health care.

## Data Availability

Not applicable.

## Abbreviations

AARP	American Association of Retired Persons
ADP	Adult Diabetic Patients
BMI	Body Mass Index
CDC	Centers for Disease Control
ESRD	End Stage Renal Disease
GDM	Gestational Diabetic Mellitus
HIP	Hyperglycemia in pregnancy
IDF	International Diabetes Federation
IFG	Impaired Fasting Glucose
IGT	Impaired Glucose Tolerance
LEA	Lower Extremity Amputations
MODY	Maturity-Onset Diabetes of the Young
NHW	Non-Hispanic Whites
NDM	Neonatal Diabetes Mellitus
ROS	Reactive Oxygen Species
SNP	Single Nucleotide Polymorphism
T1DM	Type 1 Diabetes Mellitus
T2DM	Type 2 Diabetes Mellitus
US	United States
WHO	World Health Organization
YDP	Young Diabetic Patients
